# A realist review of best practices and contextual factors enhancing treatment of opioid dependence in Indigenous contexts

**DOI:** 10.1186/s12954-023-00740-x

**Published:** 2023-03-17

**Authors:** Rita Henderson, Ashley McInnes, Ava Danyluk, Iskotoah’ka Wadsworth, Bonnie Healy, Lindsay Crowshoe

**Affiliations:** 1 University of Calgary, 2500 University Drive NW, Calgary, AB T2N 1N4 Canada; 2 Blood Tribe, AB Canada; 3 Blackfoot Confederacy, Calgary, AB T2H 2G5 Canada

**Keywords:** Medicine and health, Indigenous studies, Public health, Realist review, Community-based research, Opioid treatment, Opioid agonist therapy, Substance use, Indigenous health

## Abstract

**Objectives:**

The objective of this study was to examine international literature to identify best practices for treatment of opioid dependence in Indigenous contexts.

**Methods:**

We utilized a systematic search to identify relevant literature. The literature was analysed using a realist review methodology supported by a two-step knowledge contextualization process, including a Knowledge Holders Gathering to initiate the literature search and analysis, and five consensus-building meetings to focus and synthesize relevant findings. A realist review methodology incorporates an analysis of the complex contextual factors in treatment by identifying program mechanisms, namely *how* and *why* different programs are effective in different contexts.

**Results:**

A total of 27 sources were identified that met inclusion criteria. Contextual factors contributing to opioid dependence described in the literature often included discussions of a complex interaction of social determinants of health in the sampled community. Twenty-four articles provided evidence of the importance of compassion in treatment. Compassion was evidenced primarily at the individual level, in interpersonal relationships based on nonjudgmental care and respect for the client, as well as in more holistic treatment programs beyond biophysical supports such as medically assisted treatment. Compassion was also shown to be important at the structural level in harm reduction policies. Twenty-five articles provided evidence of the importance of client self-determination in treatment programs. Client self-determination was evidenced primarily at the structural level, in community-based programs and collaborative partnerships based in trust and meaningful engagement but was also shown to be important at the individual level in client-directed care. Identified outcomes moved beyond a reduction in opioid use to include holistic health and wellness goals, such as improved life skills, self-esteem, feelings of safety, and healing at the individual level. Community-level outcomes were also identified, including more families kept intact, reduction in drug-related medical evacuations, criminal charges and child protection cases, and an increase in school attendance, cleanliness, and community spirit.

**Conclusions:**

The findings from this realist review indicate compassion and self-determination as key program mechanisms that can support outcomes beyond reduced incidence of substance use to include mitigating systemic health inequities and addressing social determinants of health in Indigenous communities, ultimately healing the whole human being.

## Introduction

This realist review examines international literature focused on interventions for opioid dependence in Indigenous communities in countries with similar healthcare systems and colonial histories to Canada to discover activities or strategies that might be relevant in our context. The term “intervention” is commonly used in Canada to refer to programs or policies designed to respond to community- or population-level health concerns [1]. This term has different connotations elsewhere in the world, which is critically recognized and incorporated into the study out of concern that it perpetuates colonial harm [2].

While geographically and culturally diverse, Indigenous peoples internationally are affected by a common experience of colonization (i.e. community disruption, trauma, unequal access to appropriate services, and discrimination) [3–5]. Indigenous communities have variable uptake of harm reduction efforts, and we believe that a review of the international literature can help illuminate cross-cutting challenges in diverse jurisdictions as well as opportunities that may be adaptable to new spaces. In the Canadian context, the term “Indigenous” refers to First Nations, Inuit, or Métis peoples. Within the USA, the term refers to those who are Native American, Hawaiian and Pacific Islander, Alaskan, and any other Nation of reference. Within New Zealand, the term refers to Maori people. In Australia, the term encompasses Aboriginal and Torres Strait Islander peoples. In this article, we capitalize the term as well as the names of any specific nations, similar to the capitalization of other nationalities.

The realist review methodology was selected to examine which programmatic practices are most effective in the context of historical and ongoing colonization, which continues to drive substance dependence in Indigenous communities. This methodology moves beyond synthesizing literature and identifying gaps, to flesh out mid-range theory about the practices, and their contexts, that best facilitate better health outcomes for Indigenous people struggling with opioid use [6]. As such, this research begins with an understanding of the impacts of colonization on health outcomes particular to opioid dependence in Indigenous contexts, given established relationships between substance use and trauma [7], stress [8], and social disconnection [9] caused by colonization. In this context, it is likely that the opioid poisoning crisis experienced for nearly a decade across Canada is intensified in Indigenous communities.

Rates and impacts of opioid dependence in Indigenous communities are difficult to quantify. Nonetheless, provincial data from Alberta reveal that opioid dispensation and opioid-related emergency department visits and hospitalizations, as well as rates of apparent accidental opioid drug toxicity deaths, were significantly higher for First Nations (FN) clients than among the non-FN population through 2016 [10]. Rates of dispensation of buprenorphine/naloxone (i.e. Suboxone®) for FN people in the province also increased by over 3000% between 2013 and 2017, indicating interest among FN people to access opioid agonist therapies (OAT) to treat dependence [10]. While this data cannot be generalized to a single FN community, it establishes strong rationale for strengthening FN capacity to lead community-based models of care for responding to the ongoing opioid crisis and emphasizes the importance of cultural safety within environments where healthcare workers deliver care.

**Fig. 1:**
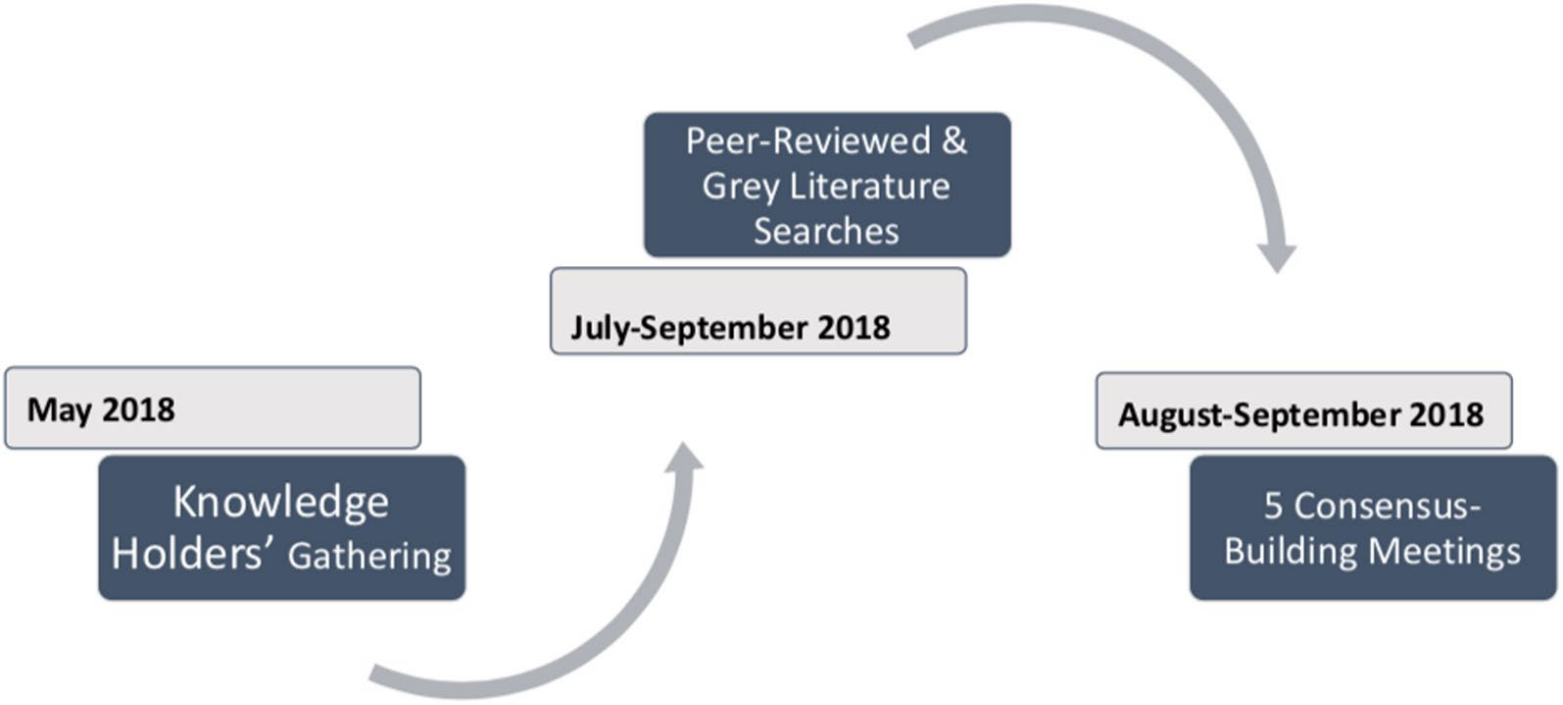
Knowledge contextualization process for the realist review

Aligned with legislation, principles, and findings from the United Nations Declaration on the Rights of Indigenous Peoples (UNDRIP) [11] and the Truth and Reconciliation Commission of Canada (TRC) [12], we affirm that the health status of Indigenous people is a direct result of historical and ongoing colonial policies and practices in the healthcare system, and that Indigenous people have the right to influence health research, policy and practices that impact them. Accordingly, this realist review was guided by Indigenous knowledge holders and Alberta FN communities affected by the opioid crisis through a knowledge contextualization process that included an initial Knowledge Holders Gathering to inform and focus the literature search and analysis, and five consensus-building working groups that included Indigenous clients and providers to improve understanding of Indigenous contexts in Alberta and appropriate programmes (Fig. 1). Despite synthesizing literature for the purpose of improving the care of FN opioid addiction in Alberta, we believe this realist review could also improve care in any international context where Indigenous populations are similarly bordered by the effects of colonization.

## Methods

This work is located within a broader opioid knowledge synthesis project initiated in response to the release of 10 Recommendations for Immediate Action by the AHS Indigenous Opioid Advisory Sub-Committee in June 2017. The project was based in partnership with the Alberta First Nations Information Governance Centre (AFNIGC), which governs research initiatives in FN communities and advances FN Ownership, Control, Access and Possession (OCAP®) principles. Our team emphasizes that connection between communities and researchers is essential to high quality and relevant research and utilizes a collaborative, consensus-building approach that centres Indigenous principles of respect for diverse perspectives and affirms a decolonizing approach to knowledge that values the community relevance of evidence yet is sufficiently systematic for policy-makers and healthcare providers. As such, a realist review guided by community is appropriate for this research.

The University of Calgary’s Conjoint Health Research Ethics Board approved components involving research with human participants (#REB18-052). The AFNIGC ensured compliance with Indigenous ethical protocols including OCAP® principles.

### Realist methodology

In this review, we use a realist methodology to synthesize evidence on current opioid use interventions to inform policy-makers and healthcare providers on best practices for opioid use programmes in Indigenous communities. A realist review goes beyond the task of descriptively synthesizing the current literature and identifying gaps in the knowledge base by allowing the reviewer to substantively identify *mechanisms*, these being how and why certain interventions are successful (previous research has provided detailed description of realist methodology [13]). Briefly, a realist review methodology incorporates an analysis of the complex interactions associated with the social determinants of health by identifying program mechanisms [14]. As such, realist reviews support the adaptation of best practices into different contexts and in doing so may better support programme planning and implementation than conventional systematic literature reviews [14].

Realist reviews begin with identification of a preliminary theory in which evidence is sought to determine in which contexts (e.g. programme leadership, community factors) a mechanism (e.g. client motivation) leads to a particular outcome (e.g. reduction in opioid use) [13]. Less explicitly identified than programme components, such as counselling, mechanisms are identified through reporting of the intervention experience by different actors involved in the programme, from managers to clients, or derived by researchers with in-depth understanding of how the programme was implemented in different contexts [13, 14]. Preliminary theory development in this realist review was guided by discussion with stakeholders [13]. The next step was to compile literature to extract evidence for (or against) the preliminary theories and refine the theories through knowledge synthesis [13]. In addition, we hosted five consensus-building meetings with clients and providers. The initial Knowledge Holders Gathering mentioned above and consensus-building meetings made up the knowledge contextualization process that guided the realist review and supported a better understanding of the community contexts.

### Knowledge contextualization process

Emphasizing that appropriate and effective programmes require connection between community members, healthcare providers, researchers, and policy-makers, we enhanced a conventional realist review with a knowledge contextualization process based in two components. First, we convened a two-day Knowledge Holders Gathering in Banff, Alberta in May 2018, which included twenty-eight Elders from FN communities from Alberta, to understand community perspectives on the opioid crisis and situate our research within key principles they identified. Second, in August and September 2018, we facilitated five consensus-building working groups made up of clients and providers to highlight the realities of Indigenous clients and the clinical experiences of healthcare providers to inform policy, funding priorities, and future research. These meetings further identified salient themes and priorities through elevating the voices of the people most closely affected by this crisis, thus adding value to the synthesis by providing contextualization to lived experiences. This contextualization process also ensures that the realist review remains relevant to Indigenous communities and empowers those working in this area to assess the evidence base in terms of appropriateness for their own community members. Following other realist reviews conducted in Indigenous contexts [14], we developed candidate theories based in these knowledge contextualization processes.

### Theory development

Though colonization impacts all Indigenous communities, how it plays out differs in unique contexts and results in different experiences for different communities. In the Knowledge Holders Gathering, Elders emphasized five key contextual factors affecting opioid dependence in their communities, including structural violence, trauma, culture, community, and experiences (full analysis presented elsewhere [15]). Structural violence outlined by knowledge holders through stories of institutional racism and stigma, has driven mistrust of western healthcare programmes and practices. Particular concerns around overprescription of opioids were highlighted, linked with stories of medications for opioid treatment such as opioid agonist therapy (OAT) that are prescribed without adequately informing clients about the goals, procedures, or risks associated with OAT. These experiences were related to processes of ongoing colonization where clients expressed concern that health professionals are wilfully not providing them with adequate information to allow them to be part of the decision-making process regarding treatment options. With these experiences, knowledge holders called for healing initiatives within communities, and that these not be restricted to abstinence-focused programmes alone but emphasize healing from the multigenerational impacts of colonization. Such healing initiatives were seen to support the decolonization of healthcare in Indigenous communities and also present a key opportunity for individuals in recovery journeys to access programmes that are culturally appropriate, support community reconnection, and promote holistic healing from the broad impacts of colonization on health and wellness.

The Knowledge Holders Gathering in Banff contextualized opioid dependence in Indigenous communities as a symptom of wider social breakdown due to historical and ongoing processes of colonization. Trauma and loss of loved ones were common experiences identified as driving dependence, a view consistent with academic research on emotional pain and stress as key risk factors in developing addiction [8]. We recognize that substance use can be a way of self-medicating in the face of colonial conditions in which people may not have the knowledge, ability, or tools to cope with stressors or pain in other, strength-based ways. Social disconnection for individuals and in communities was frequently discussed and linked to past and current government policies including residential schools, the Sixties Scoop, and the large proportion of children removed from families and placed in the child welfare system: policies separating families and extinguishing cultural practices that have ongoing implications for the health of Indigenous communities [16]. Loss of culture and community were outlined not only as drivers of the opioid poisoning crisis, but also as barriers to recovery when individuals remain isolated in recovery programs that do not resonate with them. For many individuals who have experienced substance use challenges, we were told that reconnection with culture and community are vital supports for recovery. Additionally, and also consistent with the literature [6, 17], culture and community were highlighted as important protective factors for substance use. Knowledge holders outlined their experiences with substance use in their communities, highlighting stories of resilience and deep knowledge about healing.

This knowledge and experience directed us to understand that realist review candidate theories guided by these insights must address trauma and structural violence, enhance the supports provided by culture and community, and utilize the experience of community members who have themselves overcome previous addiction. Shifting attention to mechanisms that underlie community strategies, research team members considered which mechanisms might counteract trauma and structural violence, enhance community connections, and integrate Indigenous culture and experience in treatment and harm reduction. While these are terms commonly used within healthcare and social services settings, we take treatment to refer to medical approaches for diagnosing and mitigating addiction and harm reduction to involve a broader set of policies, practices, and/or programmes that work to minimize diverse impacts associated with drug use.

Reviewing the transcripts from our gatherings for underlying themes, *compassion* and *self-determination* became evident as mechanisms to support positive outcomes of opioid programmes (e.g. greater supports for substance users, increased integration of community knowledge holders in western healthcare settings, and lower rates of drug-related medical incidences [29]). In other words, compassion and self-determination were identified as key mechanisms capable of enhancing the efficacy of opioid programmes in Indigenous contexts as they both acknowledge the underlying drivers of opioid dependence. For the purpose of this research and guided by the knowledge holders, we came to define compassion as refering to an all-encompassing kindness and consideration for life [10] and self-determination as refering to recognition of an individual or community’s ability and right to sovereignty in the decision-making process. Considering compassion and self-determination as important mechanisms to Indigenous-driven opioids programmes, we proposed two candidate theories:

- ***Candidate Theory #1***: Treatment and harm reduction models based in compassion (mechanism) for individuals and communities affected by trauma and structural violence (context) counter the stressors driving addiction and lead to saved lives, reconnected families, and people better able to reach their full potential through unhindered achievement of goals and aspirations (outcome).
- ***Candidate Theory #2***: Treatment and harm reduction models that recognize Indigenous community self-determination (mechanism) through community leadership, and culturally-based models of care integrative of community knowledge and experience with overcoming addiction (context) builds on community resilience in the face of this crisis (outcomes).

### Document selection and appraisal

**Fig. 2:**
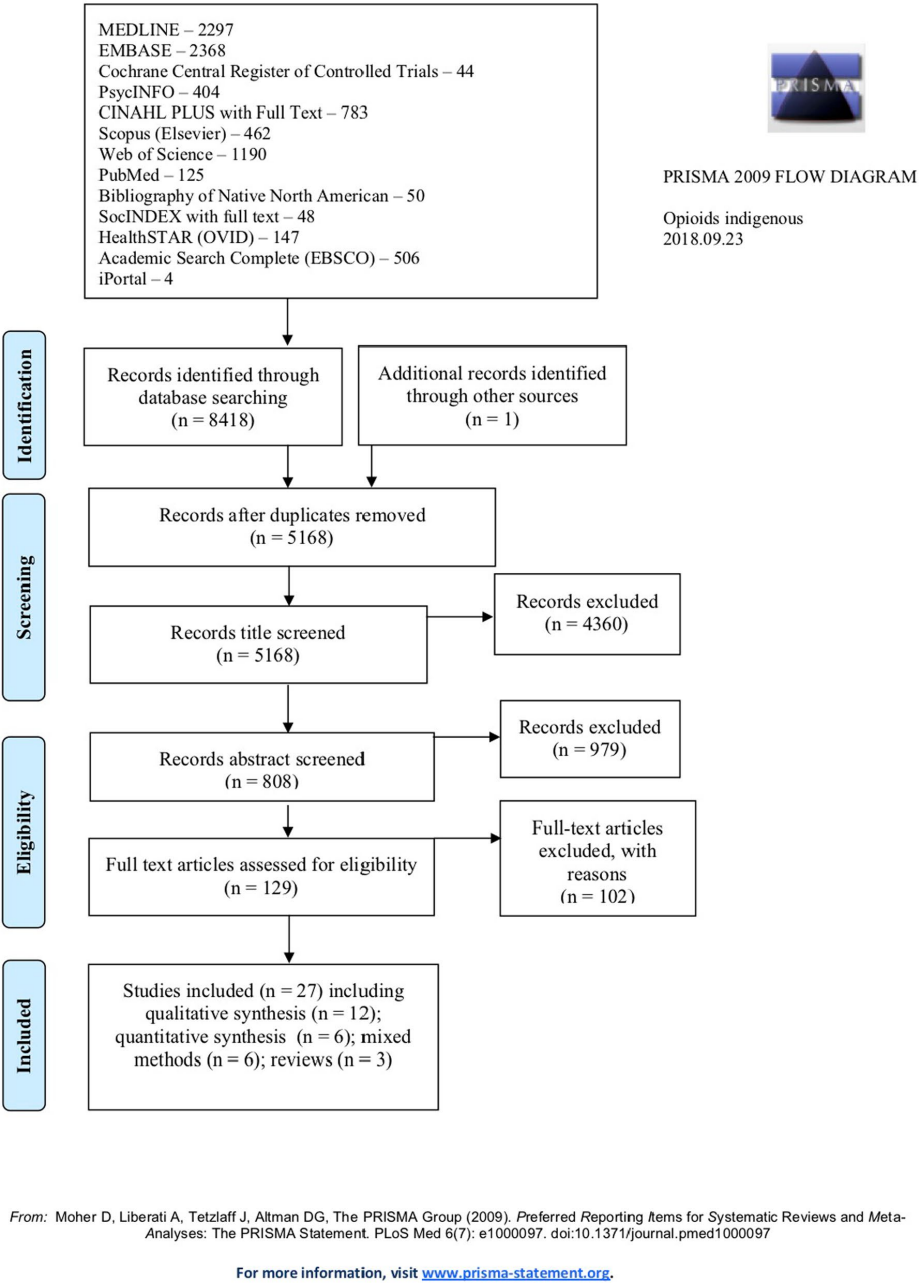
PRISMA diagram

To locate peer-reviewed literature, research team members searched a total of 13 electronic health and social sciences databases in June 2018 (see Text Box 1). Combinations of search terms used included: Indigenous, First Nations, Aboriginal, American Indian, Metis, Inuit, Pacific Islander, Aborigine, Polynesian, Alaska Native, Oceanic Ancestry Group, American Native Continental Ancestry Group, Native, Samoan, Tribe, opioids, opiates, fentanyl, street drugs, substance abuse, treatment, prevention, wrap-around supports, harm reduction, harm minimization, models of care, naloxone and/or suboxone. After removing duplicates, the articles were screened for inclusion criteria (see Text Box 2) using a three-step process (title, abstract, and full-text review) (see PRISMA diagram in Fig. 2). Of the articles in the initial literature search, 6% of the abstracts were reviewed by a second reviewer for inter-rater reliability. Of full texts assessed for eligibility, 100% were reviewed by 3 reviewers.

**Text Box 1:** Databases searched

Medline, EMBASE, Scopus, Cochrane Central Register of Controlled Trials, PubMed, PsycINFO, iPortal (Indigenous Studies Portal), CINAHL Plus, Bibliography of Native North Americans, SocIndex, Web of Science, Healthstar, and Academic Search Complete

**Text Box 2:** Search criteria

1. Describes an intervention for opioid use, prevention or treatment, or includes information on treating opioid use in the broader context of substance abuse or holistic health programs	
2. Includes information on elements of intervention success, either through qualitative methods or through quantitative methods with sufficient in-depth description of the intervention combined with quantitative methods	
3. Focuses exclusively on Indigenous populations	
4. Maintains a nonoppressive, affirming voice	
5. Research article	
6. Focuses on Indigenous communities in countries with similar healthcare systems and settler-colonial histories framing health inequities (Canada, the USA, Australia, New Zealand)	
7. English	
8. Full text available

### Data extraction, analysis, and synthesis

Articles were managed in a Microsoft Excel chart. Reason for inclusion or exclusion was tracked for all articles. Full-text screens were extracted in a more rigorous extraction framework that included a realist review extraction tool developed by Molnar et al. [18]. We extracted study information on contextual factors driving opioid use, as well as program descriptions (context), compassion and self-determination (mechanisms) and conclusions relevant to the study mechanisms including intervention outcomes, participant perspectives, and key lessons or paper conclusions (outcomes).

## Results

The initial literature search yielded 8418 results, of which 129 full-text articles were reviewed for inclusion (Fig. 2). One additional source was retrieved via a scan of article references. A total of 27 sources were identified that met inclusion criteria, including 12 qualitative [19–30], 6 quantitative [31–36], 6 mixed-methods [37–42], and 3 reviews [43–45]. Most of the articles examined perspectives or outcomes of client or community members on substance use programmes wholly or partially aimed at opioid use [19, 24–26, 31–37, 40, 42]. Some articles examined pharmacist programme outcomes [20] or care provider workshop outcomes [21, 27, 38]. Four articles examined perspectives from both clients and care providers [23, 28, 30, 39].

### Context

Contextual factors contributing to opioid dependence described in the articles often included discussions of a complex interaction of social conditions in the sampled community. The social determinants of health outlined included poverty [22, 40, 42], unemployment [22, 42], poor health [22], low education levels [22, 42], low economic development [22], incarceration [39], single-motherhood [40], domestic violence [40], racism [42], residential school experiences [22], legacies of colonialism on territorial and cultural dislocation [27], and emotional responses to colonization including grief [39], and emotional trauma and loneliness through the loss of loved ones and personal identity [43]. The social determinants of health in Indigenous contexts have been outlined in the literature; for the purpose of this realist review, the context extraction focused on program descriptions (Table 1).

**Table 1:** Context mechanism outcome table

References	Location	Study design	Study sample	Context: program description	Mechanism: compassion	Mechanism: control	Outcomes
Beckstead et al. [31]	USA	Questionnaire	American Indian/Alaska Native (AI/AN) youth (*n* = 229) from 39 tribes	Dialectical Behavior Therapy (DBT) evidence-based treatment integrated with traditional models of healing within a AI/AN youth residential treatment centre (average length of stay 120 days)	Mindfulness, a core skill taught in DBT which can include traditional models of healing such as ceremony, talking circles and smudging; program considers individual needs for quality of life (e.g. employment) alongside substance misuse treatment	Consultation with tribal leaders; local spiritual leader provided weekly spiritual practices and explained the relationships between traditional practices and the mindfulness skills taught in DBT	*Treatment outcomes*: 96% either recovered or improved using Youth Outcome Questionnaire-Self Report edition pre and post-treatment (clinically significant change criteria)
Benoit et al. [19]	Canada: Downtown Eastside Vancouver	Participant observation, semi-structured interviews, and focus groups	Aboriginal women living in Vancouver’s Downtown Eastside (DTES) (*n* = 12); 25 interviews with staff and health professionals (*n* = 61)	Vancouver Native Health Society (VNHS), an integrated and holistic (e.g. food bank) service provision and primary care setting based on integrating traditional and western approaches; Sheway, VNHS partner with a model of care based on harm reduction	Free healthcare, non-judgmental primary care and establishing trust before initiating health care; providers aim to make a connection first, work on healing	Though service providers are primarily non-Indigenous, the clinic has recruited Indigenous volunteers to support agency; participants also noted gaps in traditional healing practices	*Patient perspectives*: Women primarily indicated a need for a Healing Place, integrated and holistic health care based in respect and influence over decisions and services that impact their healing
Black et al. [32]	Australia: Winnunga Nimmityjah community	Survey	Aboriginal adult opioid dependent patients (*n* = 21)	Opioid replacement pharmacotherapy for Aboriginal patients in the ﻿Winnunga Nimmityjah Aboriginal Health Service, a community controlled primary care setting	Programs include social supports and comprehensive care within a “supportive framework” that ensures stability for patients	Community-controlled health service houses the program; community leadership in education to garner support for opioid replacement therapy and peer outreach	*Treatment outcomes*: Comparable to outcomes in mainstream programs (81% retention; no significant change in self-reported heroin use)
Campbell et al. [37]	USA: Northern Plains Region and Pacific Northwest	Surveys and Interviews conducted 1-week post intervention	American Indians and Alaska Natives (AI/AN) (*n* = 40)	The Therapeutic Education System, a web-based community reinforcement approach for substance misuse treatment completed onsite at two urban outpatient programs	Training included learning to manage negative thinking and improve self confidence	Not evident	*Participant outcomes*: 37 completed at least one module; *Patient perspectives*: Participants indicated less interest in western approaches and a desire for more culturally specific communication styles and traditional practices including spirituality
Dooley et al. [33]	Canada: NW Ontario	Retrospective chart review	Mothers and infants (*n* = 2743)	The Integrated Pregnancy Program at the ﻿Sioux Lookout Meno Ya Win Health Centre that integrates prenatal and addiction care, including OAT and tapering in the third trimester	Male partners are involved in the program and offered additional addiction treatment; respect for patient; family-centered care; postpartum care is coordinated with community-based programs to ease transitions	Includes traditional healing practices along with OAT to encourage community involvement; the authors attribute the decrease in neonatal abstinence syndrome to the local community initiatives	*Treatment outcomes*: Significant decrease in neonatal abstinence syndrome between 2009 and 2015 (*p* = 0.001); observed positive community-wide changes
Duvivier et al. [20]	USA: Southwest, Midwest, and Great Lake regions	Program description	Indian Health Service pharmacists	﻿The Prescription Drug Abuse Workgroup, Pharmacy-based interventions including responsible prescribing practices and improved access to medication-assisted treatment, comprehensive services, pharmacist-developed training for first responders	Advocate respect for the patient including supportive and nonjudgmental relationships; individualized and comprehensive treatment procedures; expansion of more comprehensive services beyond dispensation	﻿Collaborations with local governing bodies	*Care provider outcomes*: pharmacists have pledged to reduce stigma, screen for opioid use disorder, support safe prescribing, and increased access to naloxone and committed to expanding medicated assisted therapies
Gray [43]	USA	Literature Review and case study	American Indian Adolescents	Two-month, 12-step based, in-patient treatment program with weekly trauma and loss treatment groups	Trauma-informed treatment, psychological and emotional wellness to prevent relapse; safe environment for grief support; holistic healing including spirituality, cultural connection, empowerment and internal strength; family-based care (adult family members invited to attend the final week)	Traditional healing practices and individual empowerment	*Paper conclusions*: treatment must include focus on trauma and loss, connection with culture and spirituality, and healthy coping skills
Gray et al. [44]	Australia, New Zealand, Canada, and US	Editorial	Indigenous populations	N/A	Harm reduction policies; solutions that address structural drivers of health inequities; partnerships between Indigenous and non-Indigenous organizations require trust	Indigenous people must guide or be involved at all stages of research and interventions	*Key lessons*: Research and interventions must be community-based, in collaboration with communities; appropriate research, evaluation and policy (including harm reduction) must be community defined; broader structural interventions
Jumah et al. [21]	Canada: NW Ontario	Provider workshop	Service providers in Indigenous communities	Service provider workshop for care of women with opioid dependence while pregnant and postpartum, in rural and remote settings	Recommendations included increased provider education on SDOH, trauma-informed care; improved transitions between services; family-based care including keeping families intact; improved access to medicated assisted therapy to reduce risks in transporting	Recommendations included Indigenous-led programs/ partnerships with Indigenous-led programs; integration of Indigenous best practices with ‘gold standard’ medicine; funding models that encourage collaboration rather than competition between communities	*Care provider outcomes*: providers committed to Indigenous-led interventions, improved transitions in care, trauma-informed care based in Indigenous worldviews and holistic health and wellbeing, and improved access to treatment (including stable funding for Indigenous programming)
Kanate et al. [34]	Canada: NW Ontario, North Caribou Lake First Nation	Community statistics 1 year before and 1 year after the program initiation	Community-wide data	Community-based, outpatient program that integrates buprenorphine-naloxone opioid substitution and counseling from traditional healers as well as other modes of holistic healing	Community acceptance and celebration of individuals attending treatment; holistic healing; sense of community purpose	Program is managed by community nurses and healthcare providers; First Nation counselors and healers deliver culturally based and land-based healing programs	*Treatment outcomes*: Community-wide healing: Decrease in drug-related medical evacuations (-30%), criminal charges (-66.3%), child protection cases (-58.3%); increase in school attendance (33.3%); observed increase in community spirit and sense of purpose
Katt et al. [35]	Canada: N Ontario, Nishnawbe Aski Nation communities	Urine toxicology screening	FN community members aged 16–48 (*n* = 22)	Community-based 30 day suboxone tapered off or to low dose maintenance program with community-based aftercare	Aftercare programs included overall health and spiritual support	Treatment in community health centre; collaboration between off-site addiction specialists and on-site care providers	*Treatment outcomes*: 95% completed the program, 88% had no evidence of prescription opioid use in their urine toxology on day 30
Katzman et al. [38]	USA	Pre/post intervention survey	Indian Health Service clinicians (*n* = 1079)	5-h virtual education sessions for clinicians on safe prescribing and appropriate pain management	Not evident	Not evident	*Care provider outcomes*: significant increase in knowledge, self-efficacy, attitudes (p < 0.001)
Kiepek et al. [22]	Canada: Ontario, Sioux First Nation	Program overview	Sioux First Nations	Inpatient medical withdrawal support service for patients seeking abstinence in the ﻿Sioux Lookout Meno Ya Win Health Centre (SLMHC)	Holistic care (integrates physical, emotional, interpersonal, contextual factors), recognition that individual health stems from community health; trusting and responsive relationships, emphasis on patient goals and additional supports from establishing a daily routine to community leadership; follow-up care	Community-based program; integrates traditional healing including Elders in residence; individual patient goals are prioritized	*Treatment outcomes*: All but two patients successfully completed the program ﻿between December 2011 and June 2012
Landry et al. [23]	Canada: New Brunswick, Elsipogtog First Nation	Semi-structured focus groups	Three groups: professional (﻿methadone maintenance treatment program management and delivery) group, patient group, community group (*n* = 22)	Elsipogtog methadone maintenance treatment program in the Elsipogtog Health & Wellness Centre	Holistic healing, based in traditional medicine and spiritual beliefs, including life skills such as parenting practices	Community-based program, services offered in Mi'kmaq, Indigenous staff, Elders are available	*Participant perspectives*:Program considered effective at the Individual level (improved parenting practices), with some positive community impacts outlined (cleanliness, safety) but patients experienced stigma, marginalization and discrimination within the community (e.g. spiritual centers, employers) and family conflict demonstrating misinformation within the community
Lee et al. [39]	Australia: Sydney	Semi-structured interviews and surveys	Aboriginal female clients (*n* = 24) and staff (*n* = 21)	Weekly Aboriginal women's support group at an ﻿inner-city outpatient alcohol and other drug treatment service including opioid substitution treatment; format ranges from educational to informal conversation or recreational (e.g. art)	Topics vary, including treatment options and broader healing, skill building; group described as ﻿non-judgemental, offering skills-based training and broader health education including navigating systems; children welcome to attend the program; program emphasis on coming together and relaxing, providing opportunities for peer support and relationships with staff	Program encouraged client ownership of the group	*Participant perspectives*: Group members reported feeling safe, respected, supported and valued, gaining new skills, improved self-esteem and identity, more connected to services; both staff and patients reported a desire to interact more informally with each other
Mamakwa et al. [36]	Canada: NW Ontario, Sioux Lookout region	Medical record review including ﻿buprenorphine-naloxone prescribing and urine drug screening	6 First Nation communities (*n* = 526)	Community-based ﻿buprenorphine-naloxone treatment combined with traditional healing; 4 weeks of daily treatment and aftercare	﻿Inductions are in groups of 10–20 within a community-wide celebration; programs are viewed as a "welcoming back" of patients; treatment facilities are used as meeting places for healing circles; healing includes spirituality (Elder-guided, land-based aftercare)	Programs were community designed, implemented and administered; healing circles and traditional activities over formal programs	*Treatment outcomes*: Retention rates were high (72% at 18 months); urine drug screening showed high rates of negative results for illicit opioids (84–95%); *Community outcomes*: Decline in suicides in six communities; one community had ﻿declines in drug-related medical evacuations, criminal charges and child protection cases, and increase in school attendance
Marquina-Marquez et al. [24]	Canada: N Ontario	Open-ended interviews and oral story telling	Oji-Cree reserve residents, recruited through medical facilities (*n* = 35)	Community-based healing movement, including nature-based therapeutic initiatives, traditional healing methods and physical spaces for healing, often outdoors and informal	Emphasis on personal reconnection to land (place attachment integral to wellbeing), spiritual world, and identity, healing from personal traumas, maintaining family ties, love and respect for community	Grassroots movement based in culture; informal and self-paced	*Participant perspectives*: Traditional practices support healing and family/community reconnection; the grassroots/community-based nature was important for holistic healing; the informal nature allowed for individuals to engage at their own pace
Momper et al. [25]	USA: ﻿Midwestern Indian Reserva- tion	Eight Talking circles	AI adults and youth (*n* = 49)	No specific program	Not evident	One tribal council passed a resolution prohibiting OxyContin prescriptions except in terminal cases	*Participant perspectives*: Reported barriers to treatment: need for group support; worries that returning the same environment would result in relapse; lack of adequate and accessible treatment options
Radin et al. [26]	USA: 4 Washington state tribal communities	Semi-structured interviews and focus groups	Community members (*n* = 153)	General ﻿substance use, abuse, and dependence (SUAD) programs and resources across the four communities	Valued program aspects included community support for struggling individuals, family involvement in treatment, sense of identity, attention to “whole person”, life skills, and providers who are supportive	Need to address prescription drug misuse with better communication between community and healthcare providers; valued aspects of available programs included culturally-based and community driven prevention, treatment and aftercare; individualized treatment	*Participant perspectives*: valued community support, 'supportive' care providers; need for family/community wellness, adequate transition housing more supportive of recovery; need for greater community- provider collaboration, community-based and culturally-based care and healing centres; *Former patient perspectives* highlighted the need for compassion, holistic treatment (including trauma treatment), and the desire to keep families intact an important motivator
Russell et al. [45]	Canada	Scoping Review	Aboriginal	Multiple, includes a section on the National Native Drug Abuse Program (NNADAP) and community-based suboxone programs	Not evident	Scoping review focused on the successes of community-based and culturally-integrative treatment	*Paper conclusions*: Community-based treatment models have promising outcomes (high completion rates, improved abstinence, community-level improvements); more contextually and culturally appropriate treatment needed
Saylors et al. [40]	USA: San Francisco Bay Area	Program overview, staff and interviews and clinical data	Native American Women clients (*n* = 742)	﻿Women's Circle project of the Native American Health Center, focused on integrating western and Indigenous healing, holistic health;	Women’s group provides nonthreatening entry into health programs; emphasizes respect for patient/patient comfort, trauma-informed care; a nurse case manager facilitates transitions between services; holistic healing and skills training (e.g. family functioning); meeting the patient 'where they're at'	Healers from diverse communities brought in to support traditional healing for culturally diverse clients; incorporation of spirituality into counselling is guided by the patient; emphasis on Native staff	*Treatment outcomes*: Heroin use decreased by 93%; women who were using nonprescription methadone stopped after intervention; self-reported improvement in health, living conditions, increase in school attendance, decrease in involvement with criminal justice system; increase in importance of culture to participants
Srivastava et al. [27]	Canada: Ontario, Sioux Lookout First Nation	Physician interviews	Family physicians in Sioux Lookout (*n* = 18)	﻿Sioux Lookout Zone Physicians initiated an education program to reduce physician anxiety about prescribing opioids, improve the management of chronic pain, and limit the risk of addiction	Educate patients on harm reduction strategies	Physician to develop treatment agreements with patient	*Physician outcomes*: Increased awareness of addictive potential of opiates; started titrating lower potency medications for chronic pain; improved physician confidence in opioid prescribing and identification of patients with opioid dependence; increased use of treatment agreements with patients
Teasdale et al. [28]	Australia: Sydney	Interviews and focus groups	Indigenous patients and Indigenous and non-Indigenous staff (*n* = 63)	The Drug Health Service of Sydney South West Area Health Service, Eastern Zone, provides drug-related services including ﻿opioid maintenance pharmacotherapy with medication dispensed daily or every other day at the treatment centre	Staff provided after-hours support for family and community; aimed at providing therapeutic, non-authoritarian care	Priority assessment for Aboriginal clients; broad and flexible dosing hours; collaboration with Aboriginal Medical Services	*Participant perspectives*: Clients reported misinformation about methadone's health impacts, and culturally-based concerns; non-Aboriginal staff indicated a lack of cultural awareness training and appropriate care for holistic health challenges particularly to help with child protection services, heavy burden of after-hours support; *paper conclusions*: tighter partnerships with the Aboriginal community and a less formal and more welcoming service
Thomas et al. [41]	Canada: British Columbia	Semi-structured interviews and surveys including follow-up	First Nation community members (*n* = 12)	“Working with Addiction and Stress” 4-day retreat with ayahuasca-assisted group therapy NOW AN ILLICIT SUBSTANCE – REMOVE?	Primary aim was to release pain and heal through participation in ceremony and personal reflection	Involvement of ﻿local First Nations spirit-keeper	*Treatment outcomes*: Opioid use (one participant) had no change
Uddin et al. [29]	Canada: N Ontario, Eabametoong FN	Physician reflection	N/A	Northern Ontario Suboxone Support program, community-based programs; partnership between community and addiction specialists	Holistic healing in aftercare, compassionate staff	Community design and ownership is key to the program’s success	*Paper conclusion*: patients report the program has made a positive difference in their lives; author argues that Suboxone should be dispensed in the community by community nurses and trained laypeople
Venner et al. [30]	USA	Stakeholder Meeting	AI/AN Community members and AI/AN and non-AI/AN healthcare providers and agencies	National Institute on Drug Abuse stakeholder meeting to elicit feedback on medication assisted treatment in the community	Program needs outlined included holistic healing, traditional healing, patient desires to be medication free, and address systemic barriers (lack of resources in community, discrimination and inadequate care experienced outside of community)	Program needs outlined included need for better resources in community, more AI/AN care providers	*Paper conclusions*: must integrate medicated assisted therapy into traditional healing approaches and train providers to honor Indigenous ways of knowing
Williams et al. [42]	Australia: Adelaide	Discussions and electronic records	Nunga Australians (*n* = 226)	The 'Way Out' program, a holistic health program including opioid substitution	Holistic health focus on individual needs and trauma, family-based support, patient advocacy; reconciliation displays in the centre; access within a health centre to provide confidentiality; strong links with correctional services to ensure seamless transitions between services	Initiated by community, strong partnership with Aboriginal health programs in development and implementation; community members are relied upon to raise awareness about the program and ensure its success; community-based activities; Aboriginal staff; flexible appointments	*Treatment outcomes*: Program is attracting and maintaining more Indigenous clients than any previous program in the region; of those who ever accessed opioid substitution treatment, 40% are in current substitution, 10% have successfully completed, 16% transferred out, and 34% have defaulted (19% before stabilization, 15% after)

The articles included in the realist review provided examples from urban [19, 28, 39, 40], rural and remote [21, 27, 33, 35, 36], in-patient [23, 43], out-patient [34], and web-based programmes [37]. Indigenous substance use programme challenges outlined as common included a lack of stable funding [21], high staff turnover [37], a lack of cultural awareness training for staff [28], and a lack of Indigenous staff members [19]. Some articles did outline programs with Indigenous staff [23, 28].

### Mechanism: compassion

To test Candidate Theory #1, we searched program descriptions for evidence of compassion, as well as study participant (client, provider, and community member) perspectives affirming a need for compassion in treatment programmes. All but three articles [25, 38, 45] provided evidence of the importance of compassion in treatment. Broadly, compassion was evidenced at the individual level, in interpersonal relationships based on nonjudgmental care and respect for the client, as well as in more holistic treatment programmes beyond biophysical supports, such as medically-assisted treatment.

The importance of interpersonal relationships based in compassion was highlighted frequently in terms of respect for patient, therapeutic, responsive and nonjudgmental relationships, and establishing trust [19–22, 26, 28, 29, 33, 39]. In one example, providers aim to establish trust and develop relationships foremost, before initiating treatment [19]. Compassion was also in evidence through outreach workers that seek out clients who fail to attend programmes [19] or who provide after-hours care, indicating a need for systemic supports for frontline workers going beyond their job descriptions to support people [28]. An additional component of the compassion mechanism was working to ensure seamless transitions between services or into community-based care [33], including frontline staff that advocate for the individual as they transition between services [42]. Participants in one study also noted gaps in compassion as barriers to accessing services [19].

Every article that provided evidence supporting compassion as a mechanism for treatment interventions indicated a need for holistic healing programmes. Weaker examples of holistic healing within the mechanism of compassion included a web-based treatment program that included training to manage negative thinking and improve self-confidence [37] and provision of pamphlets on harm reduction strategies [27]. Most articles highlighted the importance of holistic healing. Such programmes support cultural reconnection and spiritual and emotional healing in addition to substance use treatment. Examples of programmes that integrate spirituality included Elders in residence [22], participation in ceremony [31], utilizing treatment facilities as meeting places for healing circles [36] where clients are able to share openly and informally, and land-based aftercare [36], such as fishing, taking walks, and gardening. Emotional care was profiled as including supports to heal from personal traumas [24], and ensuring safe environments for grief support [43].

Care based in compassion that supports holistic healing must focus on individual needs, which one programme defined as attention to the “whole person” [26]. This includes integrating individual client goals for substance treatment that may include a desire to be medication-free [30], which was a popular perspective considering historical and cultural contexts surrounding the relationship between Western and traditional medical practices [30]. Other broader health and wellness goals among clients included the desire to establish healthy routines and build self-esteem [22]. These include social supports and a comprehensive care framework that ensures stability for clients [32] by considering quality of life alongside substance use treatment [31]. Consideration of individual needs includes programmes that address diverse needs, such as interpersonal and contextual factors [22], empowerment and internal strength [43], parenting skills [18], and life skills or skills-based training [26]. Also important here is broader health training including support for navigating health systems [39].

Holistic healing based in compassion includes recognition of the importance of healing for families and communities beyond the individual in treatment. Holistic healing prioritizes keeping families intact [21, 24] and providing support for treatment and healing for the whole family [26, 28, 33, 42, 43]. There is also recognition that individual health stems from community health [22]. As such, several programmes integrated community healing and community support [34], for example, by holding community-wide celebrations of client inductions into treatment [36]. Supporting community health also means that there is a need to address systemic barriers to healing including a lack of resources in community, and addressing discrimination and inadequate care for individuals accessing treatment outside of their community [30].

Though compassion primarily acts as a mechanism at the interpersonal level, there was some evidence for compassion at a structural level. Models of care based on harm reduction provide evidence for compassion at a structural level [19]. Additional evidence for compassion at the structural level included mandates or provider training programmes on social determinants of health and trauma-informed care [21]. The articles indicated broad support for harm reduction policies [19, 20, 22, 23, 28, 42, 44]. Many Indigenous communities may prefer an abstinence-based approach, yet in such contexts it may be possible to increase support for harm reduction measures when it is sensitive and respectful of local needs and preferences, and is based in strong partnerships [42]. Moreover, regardless of perspectives on which models are most appropriate for addressing opioid dependence, harm reduction is an essential part of an opioid treatment conversation in order to save lives even if the ultimate aim for people with substance use disorder is abstinence, healing, and/or recovery.

### Mechanism: self-determination

To test Candidate Theory #2, we searched programme descriptions for evidence of self-determination, as well as study participant (client, provider, and community member) perspectives that there is a need for community self-determination in treatment programmes. All but two articles [37, 38] provided evidence of the importance of self-determination in treatment programs. Broadly, self-determination was evidenced at the structural level, in community-based programmes, but was also shown to be important at the individual level in client-directed care.

Strong examples of community-based care include programmes that are initiated, planned, managed and evaluated by community [21, 34, 36, 44]. Examples include community-based OAT integrated with traditional healing [23, 35, 36], a grassroots healing movement [24], and tribal council resolutions prohibiting OxyContin prescriptions except in terminal cases [25]. Structural supports for community-based programmes include funding models that encourage collaboration rather than competition between communities [21], community treatment and healing centres [35], and increasing resources in communities including more Indigenous care providers [30].

Collaborative partnerships are also important for community self-determination. Such partnerships are based in trust, with meaningful and early engagement of community leadership [21, 26, 32]. This may include collaboration between on-site care providers and off-site addictions specialists in the provision of OAT [30] or—related to the mechanism of compassion—integration of Western treatment approaches with traditional healing practices, led by community spiritual leaders [21, 22, 33, 40, 45]. The latter indicates a need for on-reserve healing spaces and community-developed healing protocols that include holistic goals [22] and community-based aftercare [21]. These programmes may be especially important when effective biophysical treatments such as buprenorphine-naloxone are not available in community [35]. For those who attend out-patient treatment centres outside of their communities, community-based counselling and aftercare programs may be important to support transitions back into community [36]. Optimally, such programmes would be based in collaborative partnerships between on- and off-site care providers and connected to longitudinal treatment plans [35, 36].

Community ownership and self-determination is important for addressing the structural inequities driving substance use, as well as mitigating mistrust [19]. In communities where abstinence-based approaches are preferred, early involvement of community leadership is important in gaining support for OAT [32]. This may include leaders educating their communities about OAT and peer outreach initiatives [32]. One evaluation emphasized the need to be sensitive and respectful of local contexts to support strong partnerships, especially in providing OAT in contexts where abstinence models are valued [42]. Community self-determination over programming initiated outside of the community also requires early inclusion of leadership in decision-making and hiring of local staff [26, 29]. In one OAT programme, the community initiated wrap-around care models, including healing circles, land-based aftercare, traditional activities, and turned inductions into community-wide ceremonies that welcomed clients back into their community and family roles [36]. Urban treatment centres prioritized hiring Indigenous staff and volunteers or relied on peer outreach to raise awareness of the programme [19, 40, 42]. In an urban treatment centre where clients have diverse spiritual backgrounds, one program invited traditional healers from the individual clients’ communities [40].

Some programmes indicated the importance of self-determination at the individual level [22]. Programmes aimed to support individual empowerment [43] and provide the individual with control over their treatment programmes [26, 27]. In one programme, individual client goals were prioritized, and personal goals may include abstaining from substances, but also holistic wellness goals important to the individual, such as exercising or spending time with family 22). Healing programmes that are informal and self-paced also support individual self-determination over their treatment [24]. One programme evaluation indicated that healing circles and traditional activities may be preferred over formal programmes [36]. One programme supported individualized treatment including client-driven incorporation of spirituality into counselling [40]. In one urban programme, the treatment centre maintained priority assessment for Indigenous clients, as well as broad and flexible dosing hours for OAT, providing elements of individual self-determination in accessing treatment [28]. Flexible appointment scheduling supports individuals with competing priorities, supporting holistic healing [42]. Also important here is strong links between services as clients transition from systems, such as correctional services, into community [42].

### Outcomes

Ten of the included articles outlined treatment outcomes, and all of these provided evidence of compassion and self-determination as mechanisms important in treatment programmes [22, 31–36, 40–42]. Of the two articles that provided results from urine toxicology screening, one study showed that 88% of participants had no evidence of prescription opioid use in their urine on day 30 of the programme (95% completion rate) [35]. The other study provided a review of a community-based OAT program that integrated traditional healing in 6 communities, where negative results for illicit opioids ranged from 84 to 95% [36]. Other evaluation methods included retention rates, which were generally high in the community-based programmes [22, 32, 36], as well as decreased rates of neonatal abstinence syndrome when examining community-wide data in the six years since the programme began [33]. One study noted that the programme, with strong evidence of compassion and self-determination in their family-based, holistic health initiative driven by community, had attracted and retained more Indigenous clients than any previous programme in the region [42], although a similar programme with evidence of compassion and self-determination noted that their programme had comparable outcomes to mainstream (western) programmes [32].

One noteworthy finding in the articles regarding outcomes is the diversity of outcome measures. Compassion as an important programme mechanism means that treatment objectives move beyond a reduction in opioid use. In Ontario, a service provider workshop indicated that keeping families intact is one goal in the management of opioid dependent pregnant and postpartum women, meaning that counselling on parenting and life skills is an important component of treatment programmes [21]. Similarly, participants in another study noted improved parenting practices as one important outcome of the programme [23]. Other holistic outcome measures included improvement in living conditions and decreased involvement in the criminal justice system [40]. When not measured specifically, participants reported outcomes including improved self-esteem, gaining new skills, and feeling safer and more connected within the care system [39]. Papers also reported holistic community-wide outcome measures [23, 33, 34, 36]. These included declines in suicides, drug-related medical evacuations, criminal charges and child protection cases, and increased school attendance [34, 36]. Also reported was an improvement in community spirit and sense of purpose [34] and an increase in community cleanliness and safety [23]. Reported provider outcome measures included decreasing stigma among pharmacists [20], commitments to Indigenous-led, trauma-informed care [21], and improved access to treatment [20, 21], and increased use of treatment agreements with clients [27].

The impacts of individual or spiritual growth beyond addiction are difficult to capture in the current studies; however, even where outcomes were not specifically measured, participants indicated a need for compassion and self-determination in programmes without necessarily naming it as such. For instance, participants in one study stated a need for a healing place with integrated and holistic healthcare based in respect, and where they would have influence over the decisions and services that impact their healing [19]. Common findings across studies included a desire for more traditional, spiritual, and informal healing opportunities [24, 28, 30, 37, 39], and accessible community-based healing centres providing opportunities for both self-determination [2, 25] and community reconnection [24].

## Discussion

Most articles included in the present study provided evidence for compassion and control as important programs mechanisms demonstrated either through measured treatment outcomes or participant perspectives on the studied programs. Our results support two mid-range theories:

- Candidate Theory #1: Treatment and harm reduction models based in compassion (mechanism) for individuals and communities affected by trauma and structural violence (context) counter the stressors driving addiction and lead to successful outcomes.

The studies outlined that compassion requires trusting, respectful relationships between care providers, holistic treatment that provides emotional and spiritual support beyond biophysical treatment, as well as educational programs that support building life skills. Compassion ensures individuals do not experience gaps in care as they transition between systems, and they remain connected with their families and communities. Broadly, compassion operates at the individual level, but can be operated at a structural level through harm reduction policies and intercultural or trauma-informed training programs for providers.

- Candidate Theory #2: Treatment and harm reduction models that recognize Indigenous community self-determination (mechanism) through community leadership and culturally based models of care integrative of community knowledge and experience overcoming addiction (context) build on community resilience and lead to successful outcomes.

The studies outlined that self-determination requires community-based care programs that are community led through all stages of initiation, planning, management, and evaluation. Woven with elements of compassion, when self-determination is operated through collaborative partnerships, these partnerships must be based in trust, as well as respectful and meaningful engagement. Self-determination supports community ownership and participation because programs are more meaningful and appropriate for the local context. Broadly, self-determination operates at the structural level with resources and support for community-based programs, but is also important at the individual level, ensuring clients have voice in their treatment programs, ensuring that treatment is appropriate and meaningful to the client.

These mechanisms have been demonstrated across the literature included in the realist review, which includes communities from across Canada, as well as from Indigenous contexts globally. Additionally, the value of compassion has been demonstrated in the international literature, in numerous provider care frameworks for Indigenous clients, such as in Educating for Equity [46], holistic and comprehensive history taking that accounts for social determinants of health including colonization [47], and in a mindfulness and spiritual approach to suicide prevention [48]. The importance of self-determination was the focus of a scoping review of substance use included in this realist review [45] and has also been demonstrated as important in Indigenous health literature including in prenatal health promotion [14], and in Indigenous health promotion more broadly [49].

### Implications

The contexts, mechanisms, and outcomes presented here have a number of implications for care providers, and policy and systems decision-makers.

Context—Public health interventions and structural-level recommendations.

1. Programme context factors such as stable funding and staff can be influenced in the short term with effective government policy
2. Contextual factors driving addiction including problem prescribing, poverty, and other social determinants of health are long-term goals, but could also be addressed with adequate program funding

Mechanisms—Avenues for care providers and programme planners.

3. Provider education on social determinants of health and trauma-informed care with an emphasis on the need for cultural humility may increase compassion
4. Effective programming requires wrap-around supports that build skills, support healing, and strengthen family and community ties
5. Programmes should be community-based in all stages: initiation, planning, management, and evaluation, or otherwise based in respectful collaborative partnerships
6. Collaborative partnerships must include full information and be community-based where possible, for instance integrating OAT into traditional health and healing centres rather than integrating spirituality into OAT

Outcomes—Considerations for researchers, care providers, and programme planners.

7. Important outcomes include reduced incidence and so-called relapse, but also holistic and individual goals, for example, increased support networks, increased sense of purpose, increased involvement in community and culture, reduced numbers of Indigenous children being removed from their families, reduced incidence with the criminal justice system
8. The international literature often focuses on the standard clinical outcomes of reduced incidence and reduced so-called relapse; however, additional outcome measures noted qualitatively in the studies included here are also valuable health outcomes that should be monitored, such as a cultural safety

### Study strengths

The realist review methodology employed here may be more relevant than systematic review methodology to Indigenous communities, leaders, policy-, and decision-makers who critique standard western approaches as reductionist and lacking adequate integration of local contexts. The integration of community perspectives and academic literature represents an important strength of this research. Additionally, the collaborative research partnership between university researchers and community partners ensures adherence to OCAP® principles and represents a key strength of this research.

### Study limitations

The analysis presented here focuses on academic literature and does not include grey literature that may provide thick descriptions and key insights on programme mechanisms. However, it should be noted that a review of the grey literature from the research team is available in a public report.^1^ This research was limited by the shortage of literature meeting our initial inclusion criteria. We were required to expand our inclusion criteria to include those studies with thin descriptions, which even so resulted in a relatively small number of studies focusing on opioid harm reduction models in Indigenous communities. As well, the literature was pulled in 2018 and, as a result, the research does not cover recent events such as the COVID-19 crisis and its affects on available programming. Because descriptions in some studies were thin, programme mechanisms had to be interpreted. This is not unusual practice for realist reviews, as program mechanisms are not always explicit [49, 50]. Future research that prioritizes thick program descriptions will better inform programmes and policy. If repeated, we would refine search terms to include “Maori”, as well as “methadone” and “buprenorphine”—common treatments for opioid use disorder.

## Conclusion

By identifying the programme mechanisms, realist reviews may support the adaptation of best practices into different contexts and as such may better support programme planning and implementation than conventional systematic literature reviews [14]. The findings from this realist review indicate compassion and self-determination as key programme mechanisms. This includes a need for care provision at the individual level that is based in respect, nonjudgmental care, individualized and holistic supports and goals, and that provides opportunities for clients to have a say in their treatment programmes. The findings indicate that at the structural level, compassion and self-determination can be maintained through policy and programming decisions that promote community-based programmes, trauma-informed care, and harm reduction. Doing so can support outcomes that go beyond reduced incidence of substance use and include mitigating systemic health inequities in Indigenous communities, addressing social determinants of health by strengthening communities, maintaining family connections, building life and job skills for individuals, and supporting a sense of purpose for individuals and communities.

## Data Availability

All data generated or analysed during this study are included in this published article and the additional file.

## Abbreviations

FN: First Nations
OAT: Opioid agonist therapies
UNDRIP: United Nations Declaration on the Rights of Indigenous Peoples
TRC: Truth and Reconciliation Commission of Canada
AFNIGC: Alberta First Nations Information Governance Centre
OCAP®: Ownership, Control, Access and Possession

## Footnotes

1 https://www.afnigc.ca/main/includes/media/pdf/digital%20reports/Healing-The-Whole-Human-Being%20FINAL%20Digital.pdf.
